# Vitamin D status and response to supplementation in very preterm infants: A prospective cohort study

**DOI:** 10.1038/s41430-026-01746-x

**Published:** 2026-04-14

**Authors:** Suphawe Wasuanankun, Prattana Rattanachamnongk, Buranee Yangthara, Sopapan Ngerncham, Ratchada Kitsommart, Punnanee Wutthigate

**Affiliations:** 1 Department of Pediatrics, Golden Jubilee Medical Center, Mahidol University, Nakhon Pathom, Thailand; 2 Pediatric Nursing Division, Department of Nursing Siriraj Hospital, Bangkok, Thailand; 3 Division of Neonatology, Department of Pediatrics, Faculty of Medicine Siriraj Hospital, Mahidol University, Bangkok, Thailand

**Keywords:** Paediatrics, Nutrition

## Abstract

**Background:**

Very preterm infants are at high risk of vitamin D deficiency (VDD), which contributes to metabolic bone disease (MBD) and other morbidities. Despite guidelines, optimal dosing remains uncertain. We determined VDD incidence and evaluated 25-hydroxyvitamin D [25(OH)D] responses to varying vitamin D intakes during the first 8 weeks of life.

**Methods:**

This prospective cohort enrolled infants born at <32 weeks’ gestation or birth weight <1500 g. Vitamin D supplementation followed institutional policy. Infants were categorized by total intake (parenteral plus enteral) during weeks 0–4: <400, 400–700, or >700 IU/kg/day. Serum 25(OH)D was measured at birth, 4, and 8 weeks. Biochemical markers and MBD screening were performed. VDD was defined as 25(OH)D < 20 ng/mL and excess (VDE) as >100 ng/mL.

**Results:**

Among 126 infants (gestational age 30 [27, 31] weeks; birth weight 1230 [950, 1570] g), 94.3% had VDD at birth. At 4 weeks, VDD persisted in 17.8% receiving <400 IU/kg/day and 6.5% receiving 400–700 IU/kg/day; vitamin D excess (VDE) occurred in 3.3% and 3.2%, respectively. At 8 weeks, normal 25(OH)D was achieved in 90.1% receiving <400 IU/kg/day and 77.4% receiving 400–700 IU/kg/day, while VDE increased to 8.6% and 22.6%, respectively. Biochemical markers remained normal; only one infant developed MBD.

**Conclusions:**

VDD is highly prevalent at birth in very preterm infants. Daily intake <400 IU/kg generally normalizes vitamin D status by 8 weeks while minimizing risk of excessive vitamin D. Higher doses may provide no additional benefit and increase risk of exceeding 25(OH)D levels.

**Clinical trial registration:**

Thai Clinical Trials Registry. Registration number TCTR20230725007. Web link: http://www.thaiclinicaltrials.org/show/TCTR20230725007.

## Introduction

Vitamin D is integral to calcium homeostasis, bone mineralization, and immune regulation. Vitamin D deficiency (VDD) is common in preterm infants, who are deprived of the third-trimester transplacental transfer that establishes fetal stores and often rely on parenteral nutrition with limited vitamin D content [1, 2]. Maternal and neonatal vitamin D status are strongly correlated; maternal deficiency therefore exerts substantial influence in preterm populations [3, 4]. Globally, VDD prevalence in very preterm infants ranges from 40% to 80% [5–8]. VDD contributes to metabolic bone disease (MBD), which may present at 6 to 8 weeks of life and, if untreated, can lead to fractures or rickets-like changes. A recent review reported MBD prevalence up to 55% in extremely low birth weight infants and 23% in very low birth weight infants [9]. Beyond skeletal consequences, VDD is associated with respiratory morbidities including bronchopulmonary dysplasia (BPD) and respiratory distress syndrome (RDS) [10–12].

Despite its clinical significance, the optimal vitamin D intake for very preterm infants remains uncertain because guidelines differ widely. The European Society for Paediatric Gastroenterology, Hepatology and Nutrition (ESPGHAN) recommends 400-700 IU/kg/day [13], whereas the American Academy of Pediatrics (AAP) suggests 200–400 IU/day for very low birth weight infants [14]. Consequently, practice varies widely across and within institutions; a recent survey in the USA reported that 77% of neonatal intensive care units administered 400 IU/day to extremely preterm infants [15].

In Thailand, data on vitamin D status in preterm infants are limited; however, vitamin D insufficiency or VDD has been reported in up to 89% of healthy term infants [16]. In very preterm infants, a recent study demonstrated a mean baseline serum 25-hydroxyvitamin D [25(OH)D] concentration of approximately 14.8 ng/mL before supplementation [17]. Despite these concerning findings, national data on the incidence and progression of VDD in very preterm infants remain limited. Moreover, substantial variability in clinical practice regarding vitamin D dosing often implemented without standardized monitoring which raises concerns about both inadequate supplementation and potential overexposure. Overexposure may result in elevated 25 (OH)D levels, which are frequently asymptomatic but may pose unknown long-term risks.

At our institution, attending physicians prescribe vitamin D supplementation at their discretion, following either AAP [14] or ESPGHAN [13] recommendations. ESPGHAN currently advocates higher doses for very preterm infants (400-700 IU/kg/day) to support bone mineralization and prevent MBD. However, baseline vitamin D status varies widely by gestational age, birth weight, and maternal vitamin D levels, which may modulate response to supplementation. These uncertainties necessitate evaluating the performance of dosing recommendations in real-world clinical settings.

Given these gaps, we aimed to determine the incidence of VDD in very preterm infants and to evaluate temporal trends in serum vitamin D levels in response to routine clinical supplementation. By examining relationship between varying levels of vitamin D intake and longitudinal biochemical outcomes, we sought to inform appropriate and safer dosing strategies for very preterm infants.

## Methods

This prospective cohort study was conducted at Siriraj Hospital, a university-affiliated tertiary care center in Bangkok, Thailand, from June 2023 to October 2024. The study was approved by the Siriraj Institutional Review Board and registered with the Thai Clinical Trials Registry (TCTR20230725007), one of the primary registries of the WHO Registry Network. Parents provided written informed consent for all participating infants. Eligible participants inborn were inborn infants delivered at <32 weeks’ gestation or with a birth weight <1500 g. The exclusion criterias were death within the first 7 days of life, major congenital anomalies, known disorders of vitamin D metabolism, or severe congenital lung and airway malformations.

### Nutrition and feeding protocol

All infants born at <32 weeks’ gestation or with a birth weight <1500 g received total parenteral nutrition with 20% SMOF lipid emulsion immediately after birth. Enteral feeding began once clinically stable, using maternal breast milk or pasteurized donor milk depending on availability. For infants weighing 1000–1800 g, feeds were started at 3 mL/kg every 3 h; those <1000 g receive 1.5 mL/kg every 3 h. Once enteral intake reached 100 mL/kg/day, breast milk or pasteurized donor milk was fortified with premature formula to achieve a caloric density of at least 24 kcal/oz. Total parenteral nutrition was typically discontinued when enteral intake of 120 mL/kg/day was tolerated. Vitamin D was administered enterally via multivitamin drops (400 IU/mL) and/or a locally compounded vitamin D2 solution (2000 IU/mL), targeting 400–700 IU/kg/day. In selected cases with markedly low baseline vitamin D levels, higher doses were used at the attending physician’s discretion, up to a maximum of 1000 IU/day. Cord blood and subsequent serum 25(OH)D measurements at 4 and 8 weeks of age were available to the clinical team throughout the study.

Maternal and infant demographic data were abstracted from medical records. Nutritional intake—including total volume, parenteral and enteral fluid intake, and caloric intake—was calculated daily. Average total vitamin D intake from birth to 4 weeks was calculated from total parenteral nutrition and enteral sources. Infants were then categorized into three groups: <400, 400–700 and >700 IU/kg/day. Daily calcium and phosphorus intakes were also recorded. Weight was measured daily; head circumference and length were measured weekly per unit protocol. Growth trajectories were assessed using the Fenton growth chart [18]. Infants with birth weight < 10^th^ percentile for gestational age were classified as small for gestational age. Clinical outcomes included necrotizing enterocolitis, diagnosed using the modified Bell’s criteria [19] and bronchopulmonary dysplasia, graded at 36 weeks’ postmenstrual age using Jensen’s criteria [20].

### Vitamin D status definitions and sampling

Serum 25(OH)D defined vitamin D status: VDD was <20 ng/mL, severe VDD was <12 ng/mL and VDE was >100 ng/mL [21, 22]. A normal 25(OH)D concentration was defined as 20-100 ng/mL [13, 21, 23]. Baseline serum 25(OH)D was measured from 1 mL of cord blood collected from each infant; subsequent samples were obtained at 4 and 8 weeks of postnatal age per the unit’s MBD screening protocol.

### Outcomes

The primary outcome was VDD incidence at birth. The secondary outcomes were temporal changes in serum 25(OH)D at 4 and 8 weeks after routine supplementation, growth trajectories across the study period, and MBD incidence. Serum 25(OH)D was quantified using electrochemiluminescence immunoassay (ECLIA) method on the Cobas® e801 Analytical Unit (Roche Diagnostics, Basel, Switzerland). Additional biochemical assessments comprised serum total calcium, albumin, phosphorus, and alkaline phosphatase levels.

### Statistical analysis

Sample size calculation was based on an assumed VDD incidence of 37% in very preterm infants [4]. We targeted enrollment of 126 infants, allowing a 25% relative margin of error and accounting for 20% dropout. Analyses were performed using SPSS version 29.0 (IBM Corp., Armonk, NY, USA) and R Studio version 2024.04 (The R Foundation for Statistical Computing, Vienna, Austria). Continuous variables were summarized as mean with standard deviation or median and (25^th^, 75^th^ percentile), as appropriate; categorical variables as frequencies and percentages. Between-group differences used independent *t*-tests or Mann–Whitney U tests for continuous variables and chi-square or Fisher’s exact tests for categorical variables. Outcomes across vitamin D intake groups (<400, 400-700, and >700 IU/kg/day) were compared using one-way ANOVA or Kruskal–Wallis tests. Generalized estimating equations evaluated longitudinal changes in serum 25(OH)D concentrations. Logistic regression identified risk factors for VDD at 4 weeks. A *p*-value < 0.05 was considered statistically significant.

## Results

**Fig. 1:**
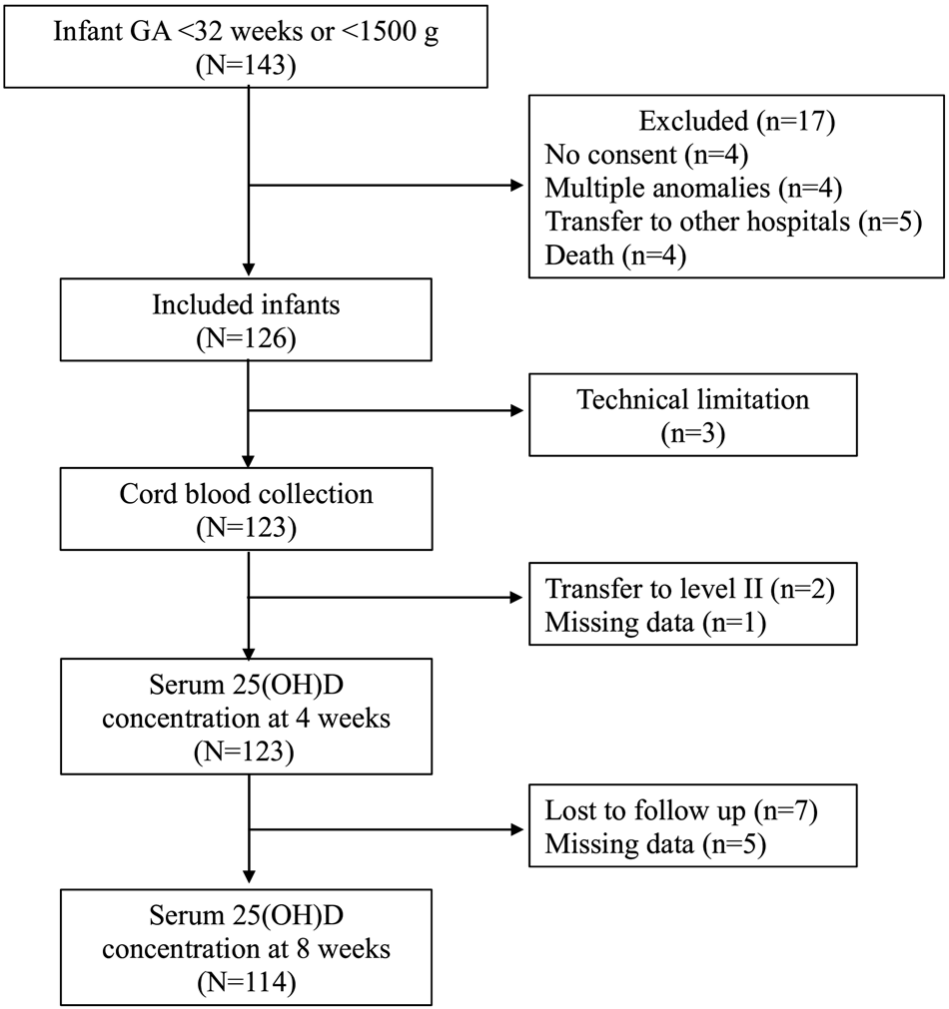
Study Flow Diagram. 25(OH)D, 25-hydroxyvitamin D; GA gestational age.

**Fig. 2:**
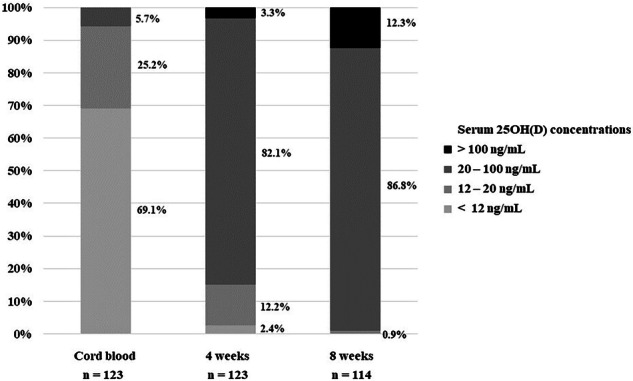
Distribution of Serum 25-Hydroxyvitamin D Concentrations at Birth, 4 Weeks, and 8 Weeks of Age. The y-axis shows percentage of infants. 25(OH)D 25-hydroxyvitamin D.

A total of 126 very preterm infants were enrolled. The median gestational age was 30 [27, 31] weeks, and the median birth weight was 1230 [950, 1570] g. Twenty-six infants (20.6%) were small for gestational age. Cord blood samples were successfully obtained from 123 of 126 infants (97.6%); three samples were unavailable because of technical limitations during collection (Fig. 1). Median cord blood 25(OH)D concentration was 9.7 [6.6, 14.2] ng/mL. VDD was observed in 116 of 123 infants (94.3%), and 85 infants (69.1%) had severe VDD (Fig. 2). Median serum 25(OH)D increased to 38.6 [25.1, 55.6] ng/mL at 4 weeks and 70.5 [47.8, 87.8] ng/mL at 8 weeks.

### Vitamin D intake groups and clinical outcomes

Based on total vitamin D intake during the first 4 weeks, infants were categorized into three groups: <400 IU/kg/day (*n* = 93, 73.8%), 400–700 IU/kg/day (*n* = 31, 24.6%), and >700 IU/kg/day (*n* = 2, 1.6%). Baseline maternal and neonatal characteristics were generally comparable across groups, except the >700 IU/kg/day group had lower gestational age and significantly lower birth weight, reflecting greater prematurity (Table 1). There were no significant differences in RDS, late-onset sepsis, NEC stage ≥2 A, or BPD among groups (Table 2). Time to initiation of enteral feeds, attainment of 120 mL/kg/day of enteral volume, and initiation of milk fortification were similar among groups (Table 2).

**Table 1:** Maternal and Neonatal Characteristics by Vitamin D Intake Group.

	Total vitamin D intake (IU/kg/day)			*p*
	<400 (*n* = 93)	400-700 (*n* = 31)	>700 (*n* = 2)	
Maternal age (years)	33.0 [28.5, 38.0]	32.0 [27.0, 35.0]	39.5 [32.0, 47.0]	0.19
Maternal BMI (kg/m^2^)	22.8 [20.6, 26.2]	21.8 [19.0, 24.8]	24.1 [23.2, 24.9]	0.26
Antenatal corticosteroid	91 (97.8)	29 (93.5)	2 (100.0)	0.48
Diabetes	21 (22.6)	3 (9.7)	0 (0.0)	0.22
Hypertensive disorders	27 (29.0)	12 (38.7)	2 (100.0)	0.07
Intraamniotic infection	22 (23.7)	8 (25.8)	0 (0.0)	0.71
Fetal growth restriction	19 (20.4)	4 (12.9)	0 (0.0)	0.51
Gestational age (weeks)	30 [28,31]	30 [27,30]	28 [24,31]	0.25
Male sex	46 (49.5)	14 (45.2)	0 (0.0)	0.36
1-min Apgar score	6 [4,8]	6 [4,8]	3 [1,5]	0.30
5-min Apgar score	8 [7,9]	8 [7,9]	7 [5,9]	0.84
Birth weight (g)	1300 [975, 1615]	1075 [860, 1370]	815 [510, 1120]	0.02^*^
Length (cm)	39.0 [34.8, 41.0]	37.0 [32.0, 40.0]	31.0 [25.0, 37.0]	0.02^*^
Head circumference (cm)	27.5 [24.9, 28.9]	26.0 [23.3, 27.8]	23.2 [19.4, 27.0]	0.04^*^

Data are presented as *n* (%), mean ± standard deviation or median [25^th^, 75^th^ percentile].

*p < 0.05 is statistically significant.

*BMI* body mass index.

**Table 2:** Clinical outcomes and nutritional parameters by vitamin D intake group.

Clinical characteristics	Total vitamin D intake (IU/kg/day) From Birth to 4 Weeks, IU/kg/day			*p*
	< 400 (*n* = 93)	400–700 (*n* = 31)	>700 (*n* = 2)	
Respiratory distress syndrome	57 (61.3)	23 (74.2)	2 (100.0)	0.24
Late-onset sepsis	43 (46.2)	11 (35.5)	1 (50.0)	0.60
Necrotizing enterocolitis, stage ≥2 A	13 (14.1)	1 (3.2)	1 (50.0)	0.07
Postnatal steroid exposure	17 (18.5)	9 (29.0)	1 (50.0)	0.29
Bronchopulmonary dysplasia	34 (37.4)	17 (54.8)	1 (50.0)	0.23
Total parenteral nutrition	84 (90.3)	28 (90.3)	2 (100.0)	0.90
Age of commencing enteral feed (hours)	3.8 [2.5, 5.5]	3.5 [2.7, 5.6]	15.1 [10.0, NA]	0.13
Duration of parenteral nutrition (days)	6.0 [4.0, 10.0]	7.0 [4.5, 9.5]	8.5 [5.0, NA]	0.92
Age of achieving enteral feeding 120 mL/kg/day (days)	6.0 [4.0, 10.0]	5.0 [4.3, 7.8]	7.5 [4.0, NA]	0.47
Age of commencing milk fortification (days)	7.0 [5.0, 11.0]	6.0 [5.0, 7.7]	8.0 [5.0, NA]	0.52
Hospital length of stay (days)	57.0 [37.5, 88.0]	69.0 [55.0, 87.0]	146 [39.0, NA]	0.13
Postmenstrual age at discharge (weeks)	39.0 [36.5, 41.0]	39.0 [37.8, 42.3]	46.5 [37.0, 50.0]	0.07
Total vitamin D intake (IU/kg/day)				
Birth to 4 weeks	236.0 [182.1, 312.0]	497.0 [452.2, 552.5]	722.5 [713.1, 730.0]	<0.01^*^
5 to 8 weeks	216.3 [168.0, 310.7]	326.1 [224.7, 417.1]	367.0 [324.0, 410.0]	<0.01^*^
Changes in Z-score from birth to 8 weeks				
Body weight	0.5 [-0.2, 0.9]	0.4 [0.3, 0.7]	0.1 [-0.1, 0.3]	0.79
Length	0.2 [0.8, -0.8]	0.1 [-0.2, 0.7]	-0.1 [-0.3, 0.0]	0.72
Head circumference	0.6 [-0.6, 1.2]	0.7 [0.1, 1.2]	-0.4 [-0.9, 0.1]	0.44
Serum biochemical bone assessment at 8 weeks				
Albumin (g/dL)	3.5 [3.3, 3.7]	3.5 [3.4, 3.7]	3.4 [2.9, 3.8]	0.92
Total calcium (mg/dL)	9.9 [9.5, 10.2]	9.9 [9.6, 10.1]	10.3 [10.0, 10.6]	0.90
Phosphorus (mg/dL)	6.2 [5.5, 6.8]	6.3 [5.8, 6.7]	4.9 [4.4, 5.4]	0.23
Alkaline phosphatase (IU/L)	327.5 [279.0, 411.0]	345.0 [278.0, 403.5]	316.0 [243.0, 389.0]	0.98

Data are presented as n (%), median [25^th^, 75^th^ percentile], or mean ± standard deviation.

*p < 0.05 is statistically significant.

“*NA*” not applicable (median cannot be calculated with *n* = 2; single value shown).

### Temporal vitamin D status by intake group and longitudinal serum 25(OH)D concentrations

**Fig. 3:**
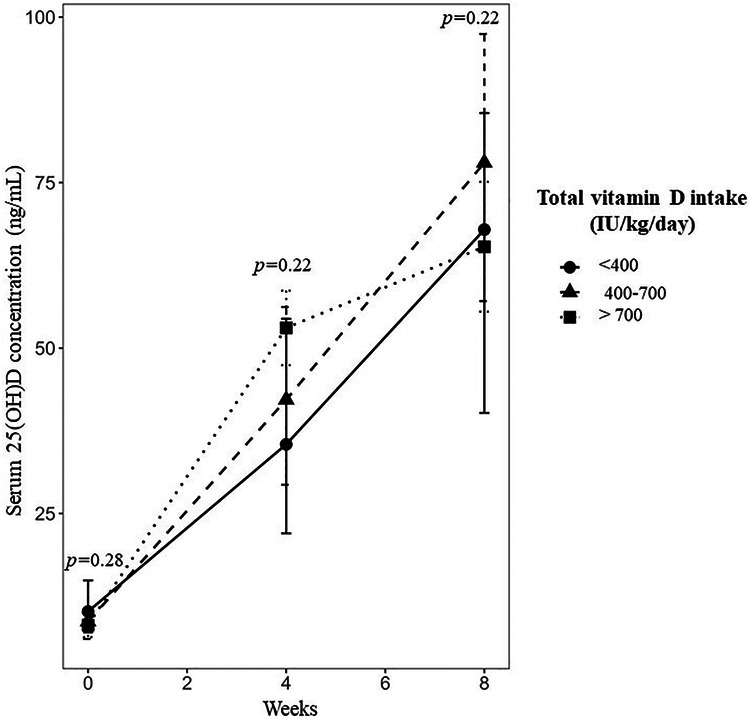
Longitudinal Serum 25(OH)D Concentrations by Vitamin D intake Group. Data presented in median [25^th^, 75^th^ percentile]. Groups are based on average total vitamin D intake (parenteral plus enteral) from birth to 4 weeks. Measurements were obtained at birth (cord blood), 4 weeks, and 8 weeks postnatal age. No significant differences were observed between groups at any time point (*p* > 0.05). 25(OH)D 25-hydroxyvitamin D.

At 4 weeks, serum 25(OH)D levels were available for 123 infants. In the <400 IU/kg/day group (*n* = 90), 71 infants (78.9%) had normal levels, 16 (17.8%) had VDD, and 3 (3.3%) had VDE. In the 400–700 IU/kg/day group (*n* = 31), 28 infants (90.3%) achieved normal levels, 2 (6.5%) had VDD, and 1 (3.2%) developed VDE. At 8 weeks, normal 25(OH)D levels were observed in 73 of 81 infants (90.1%) in the <400 IU/kg/day group and 24 of 31 (77.4%) in the 400–700 IU/kg/day group. The proportion of VDE increased to 8.6% and 22.6% infants in those groups, respectively (Fig. 2**and** Table 3). The two infants in the >700 IU/kg/day group maintained normal 25(OH)D without VDD or VDE at 4 and 8 weeks. Serum 25(OH)D increased over time in all groups. In the <400 IU/kg/day group, which received a median daily dose of 200 [100, 300] IU/day, serum 25(OH)D rose from 10.2 ng/mL at birth to 35.5 and 67.9 ng/mL at 4 and 8 weeks, respectively. In the 400-700 IU/kg/day group, cord blood 25(OH)D was 8.7 ng/mL, rising to 42.2 ng/mL at 4 weeks and 78 ng/mL at 8 weeks. Nevertheless, serum 25(OH)D concentrations did not differ among the three intake groups at any time point (*p* > 0.05) **(**Fig. 3**and** Supplementary Table 1**)**.

**Table 3:** Vitamin D status at 4 and 8 weeks postnatal age by intake group.

Total Vitamin D Intake, IU/kg/d	Serum 25-hydroxyvitamin D Concentration, ng/mL					
	At 4 wk Postnatal Age			At 8 wk Postnatal Age		
	<20 ng/mL	20–100 ng/mL	>100 ng/mL	<20 ng/mL	20–100 ng/mL	>100 ng/mL
<400	16 (17.8)	71 (78.9)	3 (3.3)	1 (1.3)	73 (90.1)	7 (8.6)
400–700	2 (6.5)	28 (90.3)	1 (3.2)	0 (0.0)	24 (77.4)	7 (22.6)
>700	0 (0.0)	2 (100.0)	0 (0.0)	0 (0.0)	2 (100)	0 (0.0)

Data are presented as *n* (%).

Vitamin D deficiency, <20 ng/mL; normal, 20–100 ng/mL; excess, >100 ng/mL.

### Growth and biochemical outcomes

Hospital outcomes are summarized in Table 2. Changes in z-scores for weight, length, and head circumference from birth to 8 weeks did not differ significantly between groups. Biochemical markers of bone metabolism; including serum calcium, phosphorus, and alkaline phosphatase, remained within normal ranges at 4 and 8 weeks, and no cases of MBD were observed in any group.

### Multivariable analysis and predictive performance

In multivariable logistic regression, male sex was associated with higher odds of persistent VDD at 4 weeks (aOR 15.69; 95% CI, 2.04–120.82; *p* = 0.008). Higher cumulative vitamin D supplementation during birth to 4 weeks was protective (aOR 0.99; 95% CI, 0.98–0.99; *p* < 0.001), as was greater cord blood 25(OH)D concentration (aOR 0.68; 95% CI, 0.51–0.90; *p* = 0.006) **(**Supplementary Table 3). The final model showed excellent discrimination, with an area under the receiver operating characteristic curve (AUC) of 0.95. When examined individually, the AUCs were 0.37 for male sex, 0.77 for vitamin D supplementation, and 0.85 for cord blood 25(OH)D. The optimal Youden’s index identified a cord blood 25(OH)D cutoff of 8.5 ng/mL for predicting persistent VDD at 4 weeks **(**Supplementary Fig. 1**)**.

## Discussion

VDD is highly prevalent in very preterm infants because inadequate third-trimester transplacental transfer limits fetal accrual of vitamin D. In our cohort, 94.3% had VDD at birth, and 69.1% met criteria for severe VDD, exceeding the 40%–80% reported in international cohorts [5–8]. Median cord blood 25(OH)D was 9.7 [6.6, 14.2] ng/mL, lower than values reported in Thailand and in international studies [5, 6, 17].

Because cord blood 25(OH)D strongly correlates with maternal vitamin D status [3, 4], these findings underscore that maternal VDD remains a major concern in tropical Thailand. Thai studies report suboptimal vitamin D levels in 76–83% of pregnant women [24, 25], reinforcing an urgent need for routine antenatal vitamin D supplementation, already endorsed by international guidelines [26, 27]. However, commercially available antenatal supplements in Thailand currently lack vitamin D, representing a critical gap in perinatal nutritional policy.

Recommendations for vitamin D supplementation in very preterm infants vary widely, ranging from a fixed dose of 200–400 IU/day [14] to weight-based 400–700 IU/kg/day [13]. A national survey of academic and level IV neonatal intensive care units in the USA highlighted substantial practice variation for extremely preterm infants, with most units administering 400 IU/day [15]. We initially followed the earlier AAP guideline of 400 IU/day for very preterm infants [14]. After ESPGHAN updated its recommendations [13], we adopted an individualized approach that allows attending neonatologists to adjust dosing based on serial serum 25(OH)D concentrations from cord blood and at 4 and 8 weeks.

At 4 weeks, 14.6% of infants remained vitamin D deficient, whereas by 8 weeks, a substantial proportion exceeded the upper threshold for optimal 25(OH)D, underscoring the difficulty of maintaining appropriate vitamin D status in this vulnerable population. In multivariable analysis, male sex independently predicted persistent VDD at 4 weeks. The mechanisms are uncertain; however, sex-related differences in neonatal outcomes may reflect variation in body composition, hormonal milieu, or vitamin D–binding protein concentrations [28–30]. Further studies should evaluate whether male infants benefit from tailored supplementation strategies. Conversely, higher cumulative vitamin D intake during the first month significantly reduced the risk of persistent VDD, although excessive intake may predispose to VDE. Cord blood 25(OH)D concentration was also protective, underscoring the importance of antenatal vitamin D transfer. Notably, receiver operating characteristic (ROC) analysis identified a cord blood threshold of 8.5 ng/dL as the optimal cutoff for predicting persistent VDD at 4 weeks. To our knowledge, this threshold has not been previously reported; however, cautious interpretation is warranted because the number of infants with persistent VDD at 4 weeks was small. Collectively, these results suggest that a uniform dosing strategy may be insufficient for all very preterm infants. Individualized supplementation tailored to baseline risk factors and guided by serial serum 25 (OH)D monitoring may therefore represent a safer, more effective approach to optimizing vitamin D status in very preterm infants.

Serum 25(OH)D increased with total vitamin D intake in a dose-dependent manner; however, the risk of VDE rose significantly at >400 IU/kg/day. In the 400–700 IU/kg/day group, 22.6% developed VDE, consistent with reports of increased risk when supplementation exceeds 600 IU/day between 2–10 weeks of age [5, 6, 31]. A fixed-dose regimen of 800 IU/day yielded no VDE in one study [17], whereas another documented only one affected infant [32]. These discrepancies likely reflect differences in dosing strategy, supplementation timing, and population characteristics. Neither our study nor prior reports found significant differences between groups in metabolic bone markers. The consistent absence of group differences suggests that serum 25(OH)D responds more rapidly to supplementation than biochemical indicators of bone health [6, 32, 33]. Early dose adjustments guided by measured serum 25(OH)D may therefore have prevent changes in bone turnover markers or overt MBD.

A recent meta-analysis demonstrated that high-dose vitamin D supplementation (≥800 IU/day) in preterm infants was associated with higher serum 25(OH)D levels and modest improvements in growth velocities, without an increased risk of vitamin D excess [34]. However, we found no significant differences in weight gain, length, or head circumference between groups. These results align with most individual trials, which show no short-term growth benefit from higher dosing. Nonetheless, specific subgroups may benefit from targeted vitamin D strategies; therefore, further research should identify which infants are most likely to show growth responses.

Our findings highlight a narrow therapeutic window for vitamin D supplementation in very preterm infants and underscore the importance of individualized monitoring of serum 25(OH)D concentrations. Strengths of this study include prospective data collection, a relatively large cohort, and comprehensive documentation of parenteral and enteral vitamin D intake throughout the early postnatal period. However, the single-center design within a specific clinical context may limit generalizability to other settings. Feeding protocols, timing of enteral fortification, and total parenteral nutrition practices all vary across institutions and countries; maternal vitamin D status also differs by geography, cultural practices, and public health supplementation policies. These factors should be considered when extrapolating our findings to other neonatal populations. Furthermore, it should be noted that while the operational cut-offs for VDD and VDE used in this study follow standard pediatric guidelines, their clinical meaningfulness in the specific context of preterm infants remains a matter of speculation and requires further validation.

## Conclusions

Vitamin D deficiency was present in 94.3% of very preterm infants at birth. Supplementation at <400 IU/kg/day generally normalized 25(OH)D by 8 weeks, whereas higher doses increased the risk of vitamin D excess without additional benefit. Although this dosing appears for most infants, individualized monitoring of serum 25(OH)D remains essential to optimize vitamin D status while avoiding both deficiency and excess.

## Supplementary information

Supplemental Material

## Data Availability

The datasets generated and analyzed during the current study are not publicly available but may be obtained from the corresponding author on reasonable request.
