# Publisher Correction: Vitamin D status and response to supplementation in very preterm infants: A prospective cohort study

**DOI:** 10.1038/s41430-026-01759-6

**Published:** 2026-05-06

**Authors:** Suphawe Wasuanankun, Prattana Rattanachamnongk, Buranee Yangthara, Sopapan Ngerncham, Ratchada Kitsommart, Punnanee Wutthigate

**Affiliations:** 1 Department of Pediatrics, Golden Jubilee Medical Center, Mahidol University, Nakhon Pathom, Thailand; 2 Pediatric Nursing Division, Department of Nursing Siriraj Hospital, Bangkok, Thailand; 3 Division of Neonatology, Department of Pediatrics, Faculty of Medicine Siriraj Hospital, Mahidol University, Bangkok, Thailand

**Keywords:** Paediatrics, Nutrition

Correction to: *European Journal of Clinical Nutrition* 10.1038/s41430-026-01746-x, published online 14 April 2026

In the sentence beginning ‘The median gestational age...’ in this article, the values ‘[24, 25]’ should have read ‘[27. 31]’.

The original article has been corrected.

