# Supplementary material for: Vitamin D status and response to supplementation in very preterm infants: A prospective cohort study
Source: Eur J Clin Nutr. 2026 Apr 14;80(7):694–9. doi: 10.1038/s41430-026-01746-x (PMC13341313; doi:10.1038/s41430-026-01746-x)
Supplement: Supplementary file 1 — Supplemental Material [file 41430_2026_1746_MOESM1_ESM.docx]

| **Total vitamin D Intake,**  **IU/kg/day** | **Vitamin D supplement,**  **IU/day** | **Median serum 25(OH)D concentrations, ng/mL** | | |
| --- | --- | --- | --- | --- |
|  |  | **Cord blood**  **(n = 123)** | **4 weeks**  **(n = 123)** | **8 weeks**  **(n = 114)** |
| < 400 | 200 [100, 300] | 10.2 [7.1, 15.1] | 35.5 [21.7, 54.6] | 67.9 [40.1, 85.6] |
| 400 - 700 | 500 [300, 620] | 8.7 [6.1, 10.7] | 42.2 [29.4, 56.2] | 78.0 [57.1, 97.5] |
| > 700 | 445 [250, 640] | 8.2 [6.3, 9.9] | 53.1 [47.4, 58.7] | 65.3 [55.5, 75.1] |
| Data are presented as median [25^th^, 75^th^ percentile]  **Abbreviations:** 25(OH)D, 25-hydroxyvitamin D | | | | |

**SUPPLEMENTAL TABLE 1.** 25(OH)D concentrations stratified Stratified by Vitamin D Intake Group Over Time

**SUPPLEMENTAL TABLE 2.** Univariable Analysis of Risk Factors for Vitamin D Deficiency at 4 Weeks

| **Parameters** | **Crude OR** | **95% CI** | ***p*** |
| --- | --- | --- | --- |
| Completed course antenatal steroid | 1.36 | 0.49 – 3.90 | 0.57 |
| Diabetes | 1.29 | 0.38 – 4.37 | 0.68 |
| Hypertensive disorder | 1.91 | 0.69 – 5.30 | 0.21 |
| Caesarean section | 3.05 | 0.66 – 14.13 | 0.15 |
| Gestational age, wk | 0.87 | 0.72 – 1.06 | 0.17 |
| Male | 3.71 | 1.14 – 12.00 | 0.03 |
| Multiple pregnancy | 0.96 | 0.32 – 2.93 | 0.95 |
| Birth weight, g | 0.99 | 0.99 – 1.00 | 0.26 |
| Late-onset sepsis | 4.23 | 1.40 – 12.74 | 0.01 |
| NEC ≥ 2A | 11.20 | 3.35 – 37.38 | <0.001 |
| Duration of parenteral nutrition, d | 1.09 | 1.04 – 1.15 | 0.001 |
| Age of commencing enteral feed, d | 1.03 | 1.01 – 1.06 | 0.009 |
| Age of achieving enteral feeding 120 mL/kg/day, d | 1.23 | 1.12 – 1.36 | <0.001 |
| Age of commencing milk fortification, d | 1.18 | 1.09 – 1.29 | <0.001 |
| Vitamin D supplement) from birth to 4 weeks, IU/day | 0.99 | 0.98 – 0.99 | <0.001 |
| Cord blood 25(OH)D concentration, ng/mL | 0.77 | 0.65 – 0.91 | 0.002 |

**Abbreviations:** 25(OH)D, 25-hydroxyvitamin D; NEC, necrotizing enterocolitis

**SUPPLEMENTAL TABLE 3.** Multivariable Analysis of Risk Factors for Vitamin D Deficiency at 4 Weeks

|  | **Total**  **(n=123)** | **VDD**  **(n=18)** | | **aOR** | **95% CI** | ***p*** |
| --- | --- | --- | --- | --- | --- | --- |
| GA, wk | 123 | 18 | 1.22 | | 0.91 – 2.10 | 0.16 |
| Male | 58 | 4 | 15.69 | | 2.04 – 120.82 | 0.008 |
| Age of commencing enteral feed, d | 123 | 18 | 1.02 | | 0.99 – 1.05 | 0.09 |
| Vitamin D supplement, birth to 4 wk, IU/day | 123 | 18 | 0.99 | | 0.98 – 0.99 | <0.001 |
| Cord blood 25(OH)D concentration, ng/mL | 123 | 18 | 0.68 | | 0.51 – 0.90 | 0.006 |

**Abbreviations:** 25(OH)D, 25-hydroxyvitamin D; GA, gestational age; VDD, vitamin D deficiency

**SUPPLEMENTAL FIG. 1** Receiver Operating Characteristic Curve for Prediction of Vitamin D Deficiency at 4 Weeks Using Cord Blood 25-Hydroxyvitamin D Concentration

**
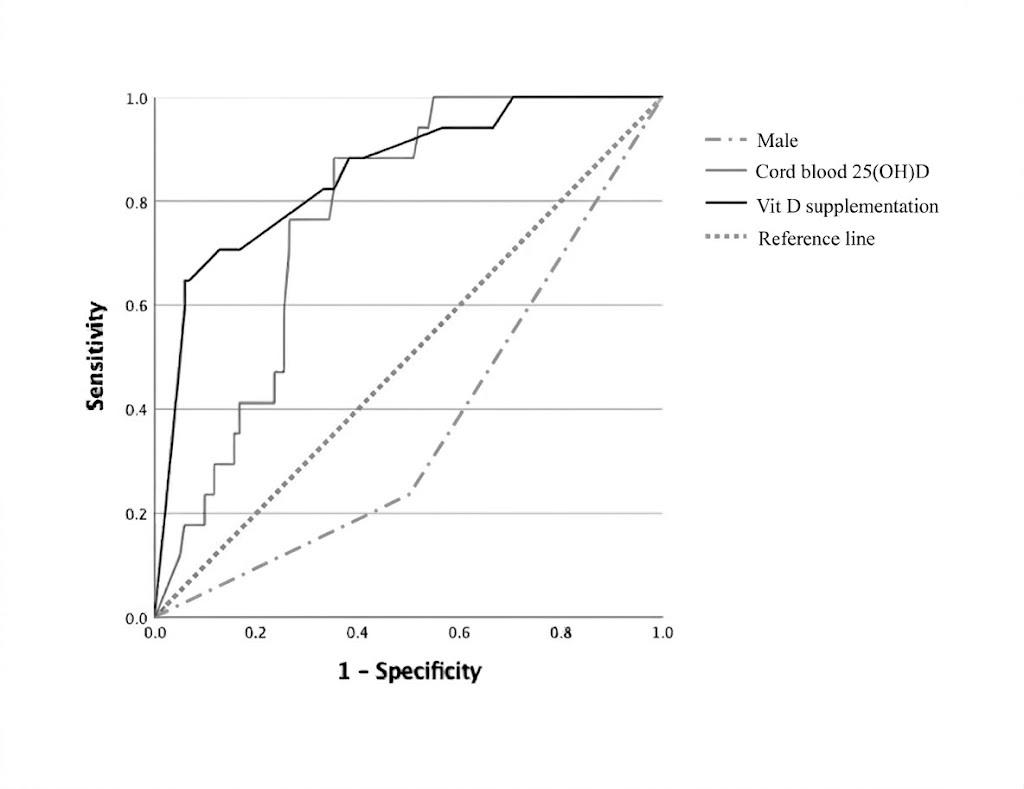
**

Area under the curve = 0.95. The optimal cutoff 8.5 ng/mL (determined by Youden’s index) yielded a sensitivity of 88 % and a specificity of 65 %
**Abbreviations:** 25(OH)D, 25-hydroxyvitamin D
